# The Frequency of Communication Between the Synovial Compartments of the Equine Temporomandibular Joint: A Contrast-Enhanced Computed Tomographic Assessment

**DOI:** 10.3389/fvets.2021.753983

**Published:** 2021-10-25

**Authors:** Karen L. Pimentel, James L. Carmalt

**Affiliations:** Department of Large Animal Clinical Sciences, Western College of Veterinary Medicine, University of Saskatchewan, Saskatoon, SK, Canada

**Keywords:** arthrography, computed tomography, magnetic resonance imaging, frequency, disc perforation, internal derangement

## Abstract

**Background:** There is conflicting evidence regarding communication between the two compartments of the equine temporomandibular joint (TMJ). Understanding the inter-relationship between TMJ compartments is critical for diagnostic and clinical management purposes.

**Objective:** To determine the frequency of communication between the discotemporal joint (DTJ) and the discomandibular joint (DMJ) of the equine TMJ in horses free of overt disease.

**Study Design:** A randomized, blinded, controlled cadaveric study.

**Methods:** Equine cadaver heads (*n* = 20), with no reported history of potential TMJ disease, were collected and stored frozen until use. Horses were randomized to the treatment group, such that Group A horses (*n* = 10) underwent arthrocentesis of the left DTJ and the right DMJ compartments, while Group B (*n* = 10) underwent arthrocentesis of the left DMJ and the right DTJ compartments of the TMJ, for a total of 40 joints. Computed tomography (CT) imaging was performed before, and after, intra-articular injection of contrast media in each head. Two observers, blinded to the treatment group, independently interpreted CT images.

**Results:** Communication between synovial compartments occurred in the left TMJ of two horses. Arthroscopic evaluation revealed that both horses had a perforation of the intra-articular disc in the region of the caudomedial fibrous expansion. Mild anterior displacement of the abnormal disc in the joint of one horse was demonstrated using magnetic resonance imaging (MRI).

**Main Limitations:** Sample size, the use of owner provided animals' history, and frozen specimens.

**Conclusions:** No physiological communication was present between the DTJ and the DMJ in the equine TMJ of the cases studied, regardless of which compartment underwent arthrocentesis. Two joints had pathological communications. These results suggest that diagnostic, and medical, treatment of intra-articular disease may be most effective when both joint compartments are injected. Furthermore, this study illustrates the value of contrast enhancement while imaging the equine TMJ.

## Introduction

The equine temporomandibular joint (TMJ) is a diarthrodial joint separated by a bi-concave, fibrocartilaginous, intra-articular disc. The larger of the two joint compartments is bounded by the mandibular fossa of the zygomatic process of the temporal bone dorsally, and the disc ventrally, forming the discotemporal joint (DTJ) (1). The smaller compartment is situated below the disc and dorsal to the condylar process of the mandible, forming the discomandibular joint (DMJ) (2, 3). There is conflicting evidence as to whether there is communication between the two joint compartments. It has been reported that the two compartments do not communicate (2, 4–6). However, one article contradicts this (7).

Primary osteoarthritis of the TMJ is a significant cause of morbidity in a large portion of the human population (8), although it is also described secondary to sepsis (9). Conversely, osteoarthritis of the equine TMJ (TMJ-OA) is most commonly reported to be secondary to joint sepsis (4, 10–13). Primary osteoarthritis with no inciting cause (trauma, sepsis, etc.) has only recently been recognized as an issue in horses where it may incite behavioral changes, poor performance, and colic (14, 15).

The most cost-effective, commonly available, least invasive technique used for the diagnosis and treatment of joint disease is arthrocentesis. The successful treatment of TMJ-OA is predicated on an extensive knowledge of joint anatomy. In humans, it is well established that the intra-articular disc completely separates the two joint compartments of the TMJ (16). This information is lacking in the horse. Therefore, the objective of this study was to assess the frequency of communication between the DTJ and the DMJ of the equine TMJ using computed tomographic arthrography, and if present to further characterize any communication using magnetic resonance imaging and arthroscopy. The hypothesis of this study was that there was no physiological communication between joint compartments in the TMJ.

## Materials and Methods

Twenty equine cadaver heads were acquired through donations of horses euthanized for causes unassociated with dental or TMJ diseases (Table 1). There were 13 geldings and 7 females, and their age ranged from 2 to 30 years old (mean 18 years, SD ± 7.8). Heads were stored frozen until use. They were thawed by immersing them in running cold water for 36–48 h prior to being utilized, and then an oral speculum was used to open the mouths maximally allowing for subsequent mandibular manipulation. Hair was clipped from the TMJ region of all heads to facilitate accurate identification of the anatomical landmarks. A numbered ear tag was placed on each head that was then randomly assigned to treatment group A or B.

**Table 1:** Description of the breeds.

**Breeds**	**n**	**%**	**Mean age**	**SD**	**Min**	**Max**
Quarter Horse	4	20	16.7	10.5	2	26
Arabian	3	15	25.7	2.5	23	28
American Paint	2	10	18	8.5	12	24
Suffolk Punch X	2	10	16	6.5	12	21
Canadian Warmblood	2	10	15	11.3	7	23
Clydesdale	1	5	20	–	–	–
Foreign Warmblood	1	5	20	–	–	–
Morgan	1	5	30	–	–	–
Percheron	1	5	12	–	–	–
Quarter Horse X	1	5	13	–	–	–
Thoroughbred	1	5	5	–	–	–
Westphalian	1	5	18	–	–	–

Heads were placed in ventral recumbency (on their mandibles), and a baseline CT series was obtained for each horse using a 320-slice computer tomography scanner (Toshiba Aquilion™ One, Otawara, Tochigi, Japan; 135 kVp, 470 mAs, 0-degree pitch using 1 mm slices). After baseline CT, heads in treatment group A (*n* = 10) underwent arthrocentesis of the left DTJ and the right DMJ compartments, while treatment group B (*n* = 10) had arthrocentesis of the left DMJ and the right DTJ compartments using a 22-gauge, 1” needle (5, 7, 17). Needle placement was confirmed by injecting between 2 and 6 ml of tap water to cause palpable and visible distention of each compartment. This was removed and replaced with a 1:1 dilution of the radiopaque contrast solution (Omnipaque, Mississauga, Ontario, CA; 350 mg/ml) with water. The total volume for light breed heads was 3 ml in the DMJ and 4 ml in the DTJ, while 5 and 6 ml, respectively, were used for the draft breed heads. After bilateral injections were completed, manipulation of the mandible through four complete masticatory cycles was performed to distribute the contrast agent throughout the synovial space. CT imaging was then repeated using the baseline protocol. Ultimately, bilateral arthrography studies were completed in each head (*n* = 20), for a total of 40 joints and 80 compartments assessed for communication.

The technician obtaining the CT images generated a random case number for each assigned ear tag number. The two investigators determining the presence of joint communication used these case numbers to access the CT images (Horos DICOM viewer, Annapolis, MA, USA) at a later date, such that they were then blinded to the treatment group. Communication was considered present if contrast agent was observed in both compartments of the same TMJ.

Characterization of any communication was performed using magnetic resonance imaging (MRI; Siemens Symphony 1.5 Tesla, Malvern, PA, USA) and arthroscopy. Three-millimeter, transverse and sagittal oblique axial T1 and spin-echo T2-weighted MRI images were obtained with the head in ventral recumbency. Furthermore, arthroscopy of the affected joints was performed using a 2.5-mm 30° arthroscope (ConMed Linvatec, Largo, FL, USA) entering both compartments (2, 5, 17). To visually confirm that the disc perforation allowed passage of joint fluid interchangeably between the DTJ and the DMJ, the arthroscope was placed in one joint compartment, while 1 ml of new methylene blue dye (Omega Laboratories Ltd., Hamon, Montreal, Canada;10 mg/ml) diluted in 6 ml of water was injected into the other compartment. The dye was subsequently removed by thoroughly flushing the joint spaces with 0.9% sodium chloride solution before switching the placement of the arthroscope and needle and repeating the dye instillation. A gross post-mortem examination of the TMJs of heads with disc perforation was performed. Histological examination of both discs of the 26-year-old Arabian horse was examined by a board-certified pathologist using routine H&E staining protocols.

## Results

**Figure 1:**
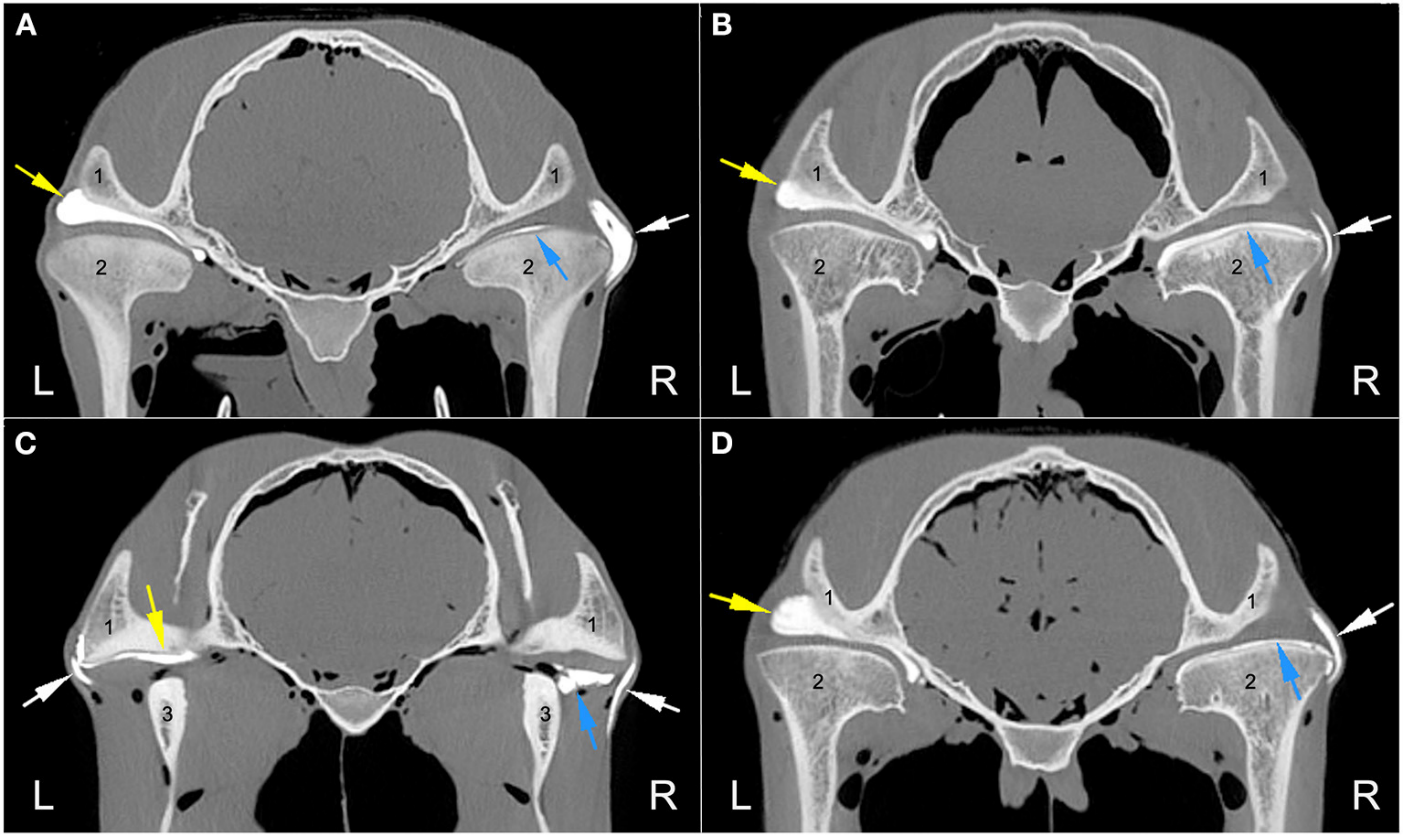
Axial computed tomography arthrography images at the level of the temporomandibular joint (TMJ) of four different heads illustrating the varying degrees of contrast extravasation. **(A)** No contrast is present outside of the left TMJ in comparison to the large amount present on the right side. **(B)** No contrast leakage from the left TMJ with mild to moderate amounts present on the right side. **(C)** Mild amount of left TMJ leakage and a mild to moderate amount on the right side. **(D)** No contrast is present outside the left TMJ with mild to moderate amounts on the right side. Arrows indicate the presence of the radiopaque contrast material in the discotemporal joint (yellow), discomandibular joint (blue), and varying amounts outside the joints (white). L, left; R, right. 1, zygomatic process of the temporal bone; 2, mandibular condyle; 3, ramus of the mandible.

**Figure 2:**
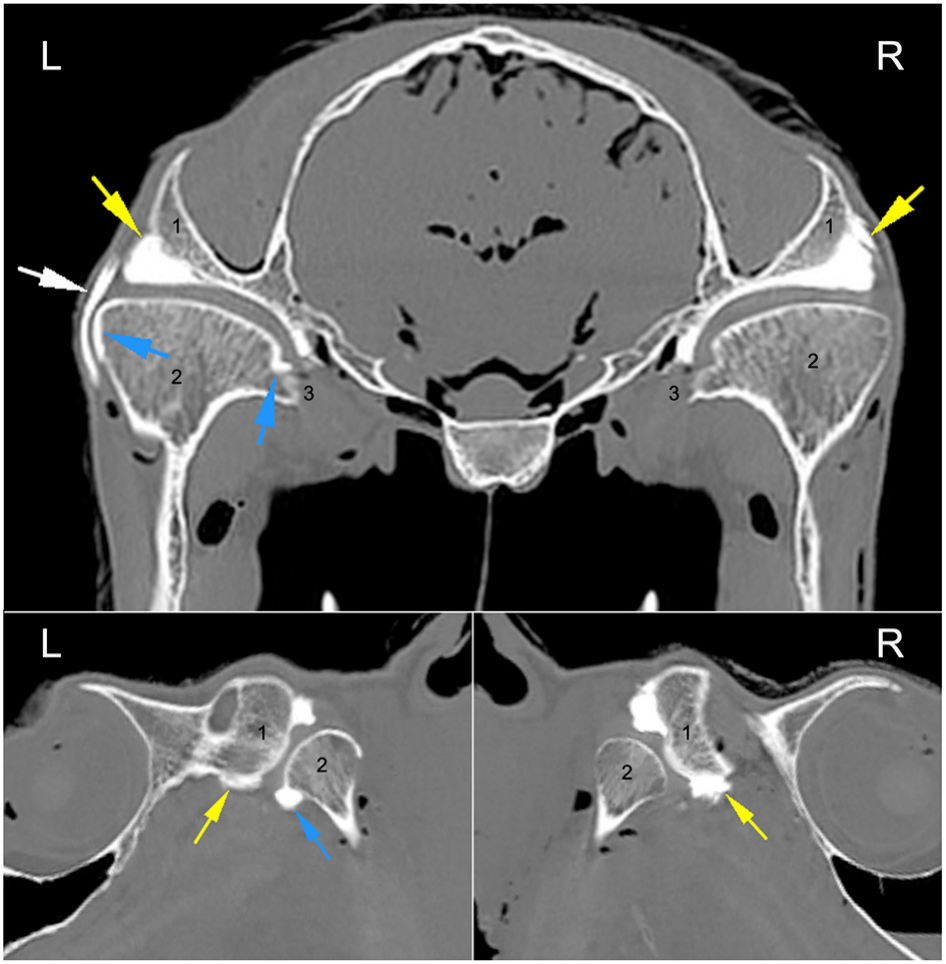
Axial and sagittal CT arthrographic images at the level of the TMJ of the 26-year-old Arabian gelding. Arrows indicate the presence of the radiopaque contrast material in the discotemporal joint (yellow), discomandibular joint (blue), and varying amounts in the subcutaneous tissues (white). L, left; R, right. 1, zygomatic process of the temporal bone; 2, mandibular condyle; 3, medial aspect of the mandibular condyle at the insertion of the pterygoid musculature bilaterally.

**Figure 3:**
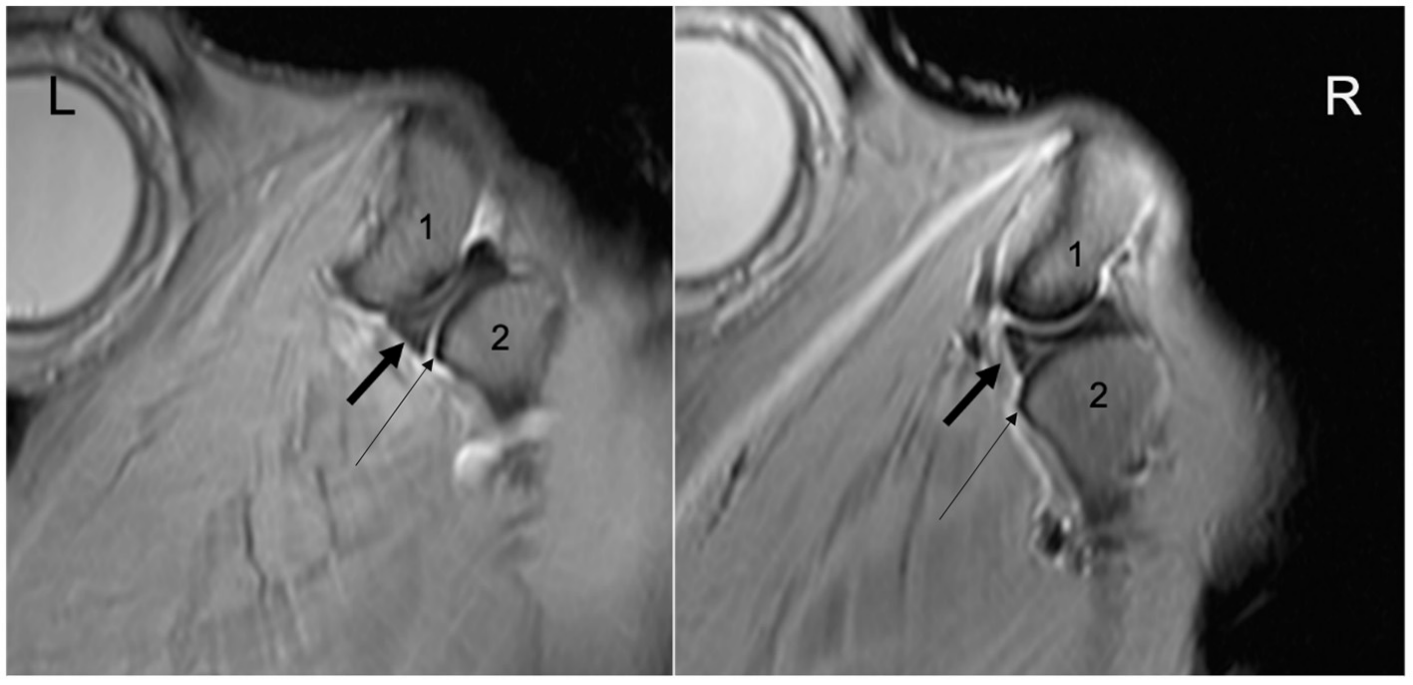
Bilateral TMJ oblique sagittal T2*W image of the 26-year-old Arabian gelding indicating mild rostral dislocation of the left intra-articular disc compared to the right. L, left; R, right. 1, temporal bone; 2, mandibular condyle. Note the difference in relative position between the rostral margin of the intra-articular discs (thick black arrow) and the rostral margin of the mandibular condyles (thin arrows).

Arthrocentesis, and subsequent imaging, of all joint compartments was successful. There were varying degrees of extravasation of contrast agent in the majority of horses (Figure 1). Communication between compartments occurred in the left TMJ of two 26-year-old (one Arabian and one Quarter Horse) geldings (Figure 2). The former horse had the DMJ injected originally, whereas the latter occurred after injection of the DTJ. The oblique sagittal T2-weighted MRI images revealed that the left intra-articular disc was mildly displaced rostrally compared with the right in the Arabian horse (Figure 3). No abnormalities were noted in the Quarter Horse.

**Figure 4:**
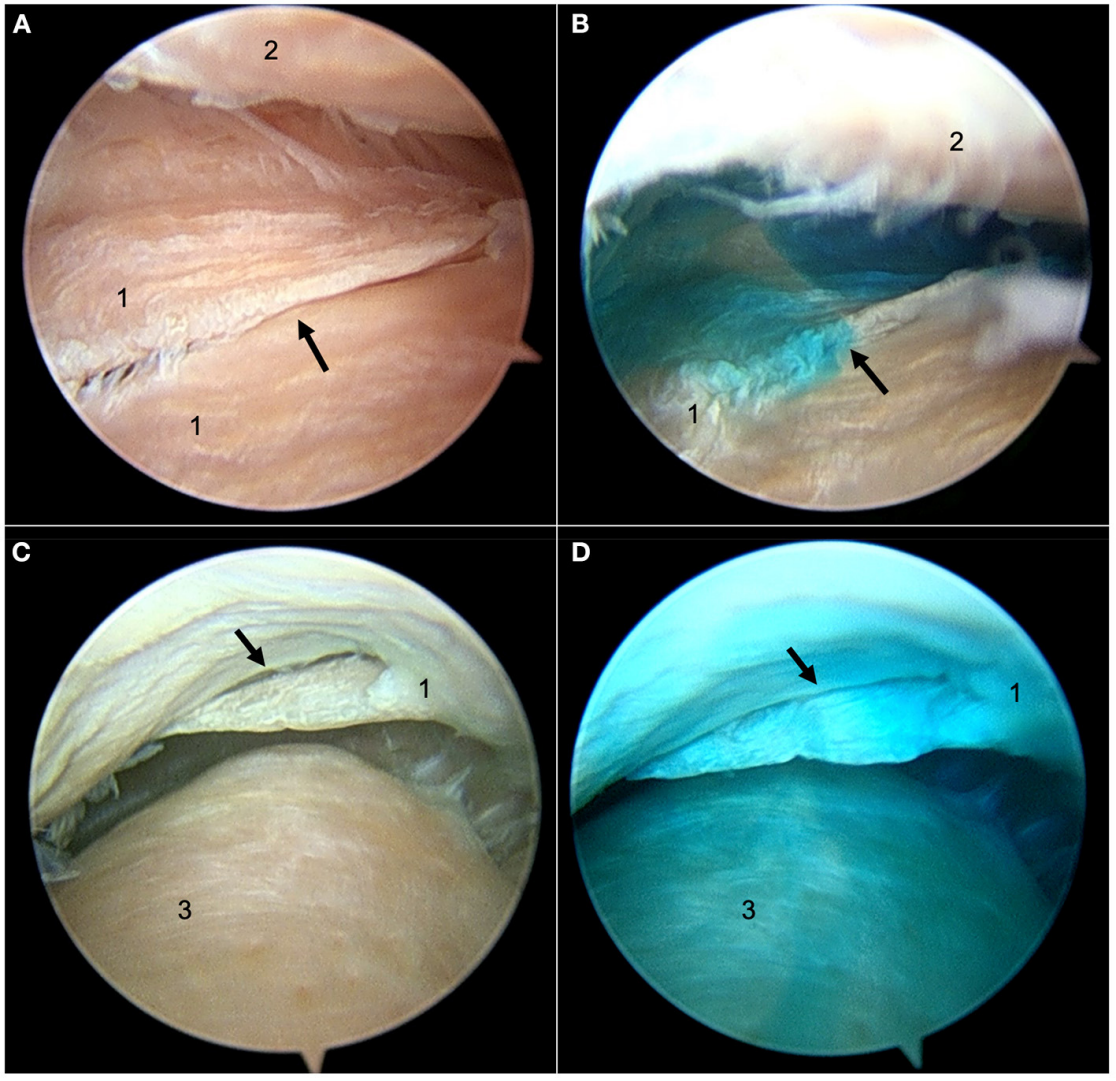
Still arthroscopic images from the left TMJ of the 26-year-old Arabian gelding. **(A)** The perforation (black arrow) in the intra-articular disc viewed from the discotemporal joint (DTJ) before new methylene blue dye injection. **(B)** New methylene blue dye passing through the perforation up into the DTJ after being injected into the discomandibular joint (DMJ) below. **(C)** The perforation (black arrow) viewed from the DMJ before dye injection. **(D)** Dye in the DMJ after new methylene blue dye injection into the DTJ. 1, intra-articular disc; 2, articular eminence of the temporal bone; 3, mandibular condyle.

Bilateral arthroscopy revealed minimal superficial fibrillation of the articular cartilage in the right TMJ of both heads and perforations of the intra-articular discs, with a similar degree of cartilage fibrillation of the left TMJ. The perforation was located in a rostro-caudal orientation in the Arabian head and in a rostro-lateral to caudo-medial orientation in the Quarter Horse head. Both were identified in the caudomedial fibrous expansion of the disc (1). Evaluation of the flow of dye confirmed that the defect had not created a one-way valve effect (Figure 4).

Gross post-mortem examination of the TMJs of horses that had disc perforation confirmed the fibrillation of the fibrocartilage of both joint compartments with no apparent difference between the joints with disc perforation and those without. Histological examination of the discs found that the tissue adjacent to the perforation had a higher proportion of poorly staining cells and rare empty lacunae than the same region of the unaffected disc, which was consistent with necrosis. Additionally, the collagen bundles adjacent to the perforation were frayed, or separated.

## Discussion

The objective of this study was to assess the frequency of communication between the DTJ and the DMJ of the equine TMJ. The results of the study described above confirmed our hypothesis in that there was no communication between the two compartments in normal horses. Our results differ from those of Rosenstein et al. (7) who reported communication between the joint compartments in normal horses. In that report, TMJs were assessed using radiopaque contrast and new methylene blue dye injection. Despite this publication, numerous other publications (2, 4–6, 17), using a variety of different protocols, have not described a communication between the DMJ and the DTJ. Unfortunately, the former authors (7) do not report any further anatomical or histological investigation to determine the cause or location of the communicating channel(s). It is possible that the consistency of the latex material used in some studies (2, 6) relative to that of injectable contrast material led to differing results; however, both Rosenstein et al. (7) and Weller et al. (5) used similar material and reported different results using different imaging methods.

The previously reported morphological variations in the equine DMJ capsule may have represented avenues of joint communication (17). As such, the current study used a 1:1 dilution of the commercial contrast agent with water, reducing the viscosity. The theory was that a less viscous solution would facilitate flow throughout the joint space and pass through any small points of communication. Extravasation of the contrast material occurred in the majority of injected joints (Figure 1), demonstrating that this goal had been achieved. Despite filling the injected joints with contrast material, the only TMJs confirmed to have joint compartment communication were those with disc perforations. Extrapolation of our findings to the diagnostic process, and treatment, of equine TMJ disease would suggest that medicating a single compartment may not result in the desired effect. Pharmacologically significant concentrations of injected medication may diffuse across the thin tissue of the intra-articular disc or joint capsule, as occurs in hock and stifle joints of horses (18, 19), but further studies are needed to confirm whether this clinically useful effect occurs in the TMJ.

There are reports of some diseased (septic) equine TMJs having destruction of the intra-articular disc and subsequent communication between compartments (4, 12); however, the authors are only aware of a single previous description of a disc perforation associated with primary osteoarthritis (20). Involvement of the intra-articular disc is common in humans and dogs with TMJ disease (21, 22), where characteristic defects occur in specific locations. In contrast, substantially less is known about the interrelationship between the disc and TMJ-OA in the horse. A recent paper illustrated regional differences in the compressive stiffness of the disc in horses and reported that a decrease in stiffness was associated with joint disease and cartilage erosions on the condylar surfaces of the joint (23). Interestingly, there was superficial fibrillation of the articular cartilage on the condylar surfaces of the TMJs with disc perforation in the current study, but overt erosion was absent. Given a recent finding that 15% of horses had mineralization of the intra-articular disc noted on CT examination (24), it is possible that substantially more horses have clinically silent TMJ disease associated with, or compounded by, disc disease. In the aforementioned study, disc mineralization did not appear to be associated with other abnormalities and seemed to be primarily associated with horse age. While concurrent mineralization and perforation of the intra-articular discs was not noted in the current study, it is interesting to note that disc perforation occurred in older horses. It is possible that age-related changes in the properties of the caudomedial fibrous expansion of the disc predisposed it to perforation, especially given the similar location and positioning of the pathology in both affected horses. Of course, iatrogenic damage may have been a cause of disc perforation especially given that the defects in the equine discs did not look like those classically seen in humans and dogs. Despite this difference, an iatrogenic cause was considered unlikely given the anatomical position and orientation of the perforations, as well as the lack of damage to the articular surfaces. Ultimately given that the authors can only find one picture of disc perforation in primary TMJ-OA in horses, and only two horses in the current study had disc perforation, much more research is needed before any conclusions as to the etiology and consequence of this condition can be extrapolated to the horse population at large.

A retrospective assessment of the baseline CT images of the TMJs in which a disc perforation occurred in the current study revealed no apparent abnormality of the disc. It may be that the angle and location of the perforation in these horses precluded identification of the pathology without contrast enhancement. If this is the case, then it is possible that disc perforation occurs more commonly in the horse than is reported, and that the same pathology may have been identified in earlier publications if contrast-enhancement had been part of the standard imaging protocol.

Internal derangement (an abnormal position of the disc) is a common finding in human TMJ-OA (25, 26). In our study, both horses with a perforation of the disc underwent MRI examination and one had a slight rostral dislocation of the perforated left disc when compared to the right. The perforation could not be visualized. It is possible that this small difference is simply normal variation, especially given the orientation of the disc perforation, the fact that there is only a single report describing the MRI anatomy of the equine TMJ (27), and that disc displacement has been reported in up to 35% of asymptomatic human volunteers undergoing MRI of their TMJs (28). However, it may also represent an example of the internal derangement reported in other species. More research is needed to evaluate the normal position of the equine disc using MRI and to further understand the inter-relationship between disc position and joint disease in the horse.

Limitations of the current study include the lack of a clinical history (specifically as it relates to mastication and the TMJ); the lack of an oral examination and assessment of temporal and masseter mass to attempt to quantify the clinical effects of the intra-articular disc perforations; and the use of thawed, previously frozen cadaver heads. However, given that horses with TMJ abnormalities may not show any localizing clinical signs (15, 29), it is possible that the horses with disc pathology had painful, or had altered mastication, which was not appreciated by the caregivers. Furthermore, the objectives of the study were to report on the frequency of TMJ joint compartment communication in our population of horses, and not to determine the effect of that pathology. Finally, while we recognize that using frozen cadaver material is not ideal, the lack of histological difference between the perforated and normal disc suggests that the method of storage did not contribute to the identified pathology.

## Conclusion

No physiological communication between joint spaces was present in our population, regardless of which compartment underwent arthrocentesis. Two horses had perforations of the intra-articular disc of the left TMJ resulting in contrast material being present in both joint compartments. Neither of these defects were identified using computed tomography before, or after, contrast enhancement. The presence of the contrast agent in both joints led to further investigation that resulted in the diagnosis. The results of the present study may be extrapolated to suggest that both compartments of the equine TMJ should be medicated, whether for diagnosis or treatment, to deliver the best outcome in clinical cases. Furthermore, advanced imaging without contrast-enhancement may result in an incomplete assessment of the TMJ.

## Data Availability Statement

The raw data supporting the conclusions of this article will be made available by the authors, without undue reservation.

## Ethics Statement

Ethical review and approval was not required for the animal study because the research was performed on cadaver tissues and thus exempt from ethical review and approval.

## Author Contributions

KP contributed to the study design and execution, data collection, interpretation and analysis, and manuscript preparation. JC contributed to the study concept, design and execution, data interpretation, and manuscript preparation. Both the authors approved the final manuscript.

## Funding

This research was supported by a University of Saskatchewan—Accountable Professional Expense Fund. The funding source had no role in the study design, specimen collection, analysis and interpretation of data, writing of the manuscript, or decision to submit the manuscript for publication.

## Conflict of Interest

The authors declare that the research was conducted in the absence of any commercial or financial relationships that could be construed as a potential conflict of interest.

## Publisher's Note

All claims expressed in this article are solely those of the authors and do not necessarily represent those of their affiliated organizations, or those of the publisher, the editors and the reviewers. Any product that may be evaluated in this article, or claim that may be made by its manufacturer, is not guaranteed or endorsed by the publisher.

## Abbreviations

TMJ: temporomandibular joint
DTJ: discotemporal joint
DMJ: discomandibular joint
CT: computed tomography
MRI: magnetic resonance imaging
OA: osteoarthritis
TMJ-OA: temporomandibular joint osteoarthritis.
